# Case Report: Extended survival in KRAS-G12V NSCLC with leptomeningeal metastasis through integrated intrathecal chemotherapy and systemic therapies

**DOI:** 10.3389/fphar.2025.1632369

**Published:** 2025-10-22

**Authors:** Yun Chen, Zhiquan Qin, Luying Zhan, Xinchang Guo, Koji Fukuda, Qihao Zhou

**Affiliations:** ^1^ Cancer Center, Department of Medical Oncology, Zhejiang Provincial People’s Hospital, Affiliated People’s Hospital, Hangzhou Medical College, Hangzhou, Zhejiang, China; ^2^ The Second Clinical Medical College, Zhejiang Chinese Medical University, Hangzhou, China; ^3^ Internal Medicine Department, Pingyang Changgeng Yining Hospital, Wenzhou, China; ^4^ Division of Innovative Cancer Control Research, Cancer Research Institute, Kanazawa University, Kanazawa, Japan

**Keywords:** leptomeningeal metastasis, non-small cell lung cancer, KRAS-G12V mutation, intrathecal chemotherapy, ventriculoperitoneal shunt, Ommaya reservoir, case report

## Abstract

Leptomeningeal metastasis (LM) is among the most severe complications in lung cancer patients, particularly for those without targetable gene mutations, who typically survive just 1–4 months. We present the case of a 68-year-old man with non-small cell lung cancer (NSCLC) and LM (pT1cN0M1b, stage IVB) whose primary lesion was early-stage with no other distant metastases. Genetic testing identified only a KRAS-G12V mutation. After neurological symptoms progressed following one cycle of pemetrexed, bevacizumab plus platinum-based chemotherapy, the patient underwent ventriculoperitoneal shunt placement and Ommaya reservoir implantation. Treatment with intrathecal pemetrexed via the Ommaya reservoir, combined with intravenous tislelizumab and carboplatin, resulted in 12 months of progression-free survival. For subsequent central nervous system progression involving both brain parenchymal metastasis and LM, we administered whole brain radiotherapy followed by second-line intrathecal thiotepa via Ommaya reservoir alongside tislelizumab and bevacizumab. This achieved continued shrinkage of brain lesions and neurological improvement. After ten cycles, thrombocytopenia necessitated switching to intrathecal methotrexate. Remarkably, the patient has survived nearly 29 months while maintaining good performance status and quality of life - to our knowledge, one of the longest reported survival for an NSCLC patient with LM harboring KRAS-G12V or other non-targetable mutations. This case suggests that combining ventriculoperitoneal shunt with Ommaya reservoir-delivered intrathecal chemotherapy may represent an effective therapeutic approach for LM patients.

## 1 Introduction

Leptomeningeal metastasis (LM) refers to the spread of malignant cells to the meninges, including the pia mater and arachnoid mater, as well as the subarachnoid space and other cerebrospinal fluid (CSF) spaces. When these cells originate from a solid tumor, the condition is known as leptomeningeal carcinomatosis (LCM) or carcinomatous meningitis. About 5% of patients with malignant tumors will develop LM, among which lung cancer, breast cancer, and melanoma are the most common (Ozcan et al., 2023). In lung cancer, the prevalence of LM has been reported to be 10%–26% (Jafari et al., 2024). The lifespan after LM diagnosis has a median survival time of approximately 1–4 months (Jafari et al., 2024; Lamba et al., 2021). Unfortunately, there is a lack of standardized treatment methods for LM. Especially for non-small cell lung cancer (NSCLC) patients who have negative driver genes, as well as those who develop LM after failing system targeted drug treatment. Intrathecal chemotherapy (IC) is a successful treatment for patients with LM from NSCLC (Wu et al., 2016). The efficacy of IC in LM has been explored in some small sample clinical studies, and some results have been achieved (Fan et al., 2024; Li H. et al., 2023; Fan et al., 2021; Miao et al., 2020; Ju et al., 2016). Intraventricular administration via an implanted Ommaya reservoir is the preferred intrathecal route for drug administration (Jafari et al., 2024). It can avoid the drawbacks associated with lumbar puncture injections, such as patient discomfort, lumbar puncture syndrome, puncture failure or bleeding, local chemical arachnoiditis, insufficient medication in cerebrospinal fluid, single-dose non-fractionated administration, and higher neurotoxicity. However, using only an Ommaya reservoir may potentially lead to adverse effects, such as cerebrospinal fluid leakage, local infection, and non-healing wounds, especially, unstable intracranial pressure (ICP), which can lead to a rapid decline in the patient’s physical condition. The ventriculoperitoneal shunt (VPS) perfectly addresses the drawbacks associated with simple Ommaya reservoir implantation (ORI). Here we report a case of a 68-year-old man diagnosed with lung adenocarcinoma (LUAD, one of NSCLC) complicated by LM, featuring a Kirsten rat sarcoma (KRAS) p. G12V mutation. The patient demonstrated significant clinical improvement with combination therapy, achieving complete CSF cytological clearance. At almost 29 months after the LM diagnosis, he maintains excellent functional status. To our knowledge, this represents the longest documented survival in NSCLC patients with LM harboring KRAS-G12V or other non-targetable driver mutations, marking a notable advancement in managing this prognostically challenging condition.

## 2 Case presentation

### 2.1 Clinical findings and diagnosis

**FIGURE 1:**
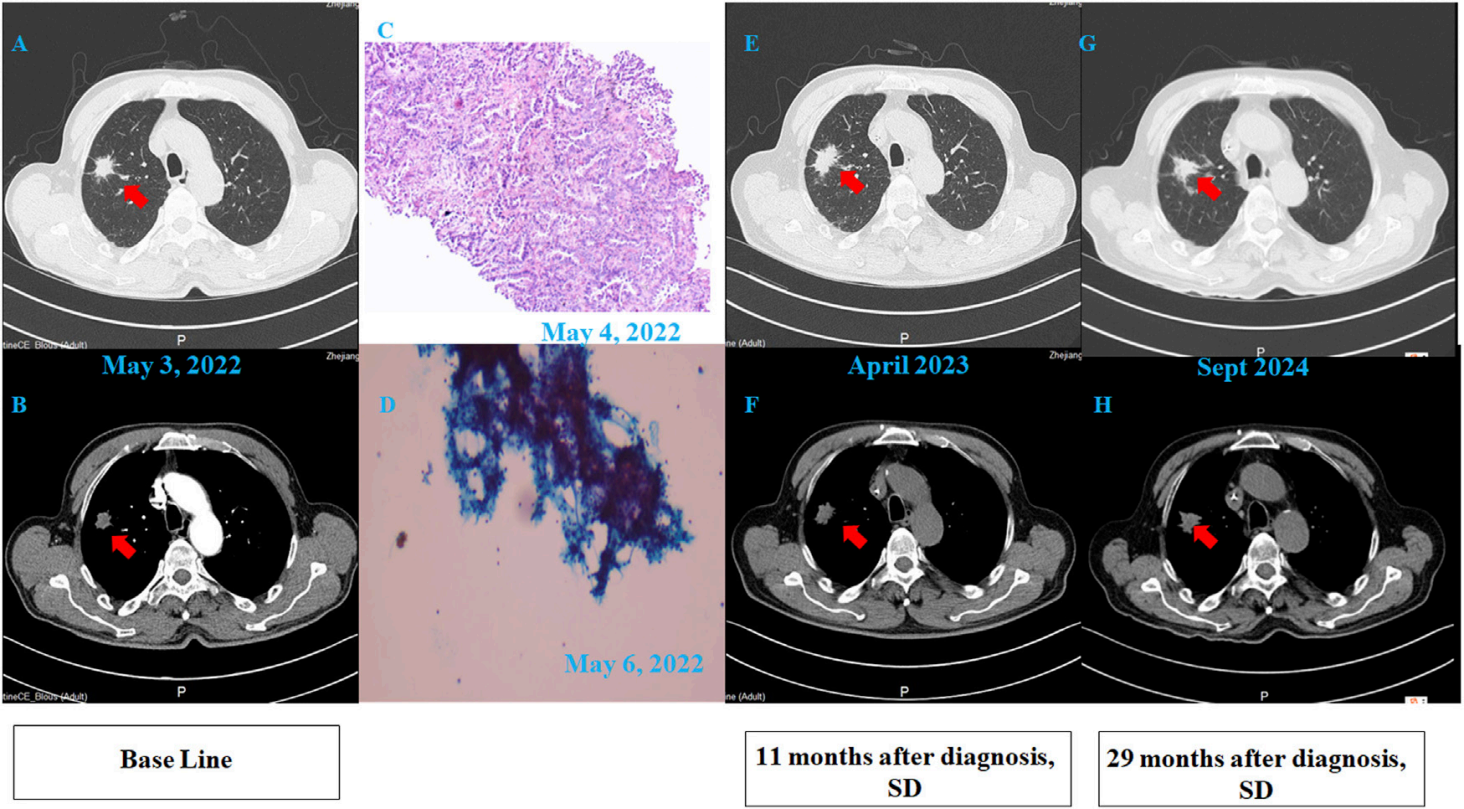
Clinical data of the patient. **(A,B)** Pulmonary CT scan revealed an irregular nodular shadow (20 × 22 mm) in the apical segment of the right upper lobe, with lobulation and spiculation. The lesion showed unclear boundaries and mild enhancement on contrast imaging. **(C)** Pathology: “Lung-mass needle biopsy” – adenocarcinoma. Immunohistochemistry: PD-L1 assay on DAKO 22C3 platform; TPS = 10%. (100×). **(D)** Cerebrospinal fluid (CSF) smear pathology revealed clustered atypical cells, highly suspicious for malignancy. (100×). **(E,F)** Follow-up CT scan at 11 months post-diagnosis showed no significant interval change in the right upper lobe nodule. **(G,H)** Follow-up CT scan at 29 months post-diagnosis demonstrated stable disease (SD), with the nodule remaining unchanged.

**FIGURE 2:**
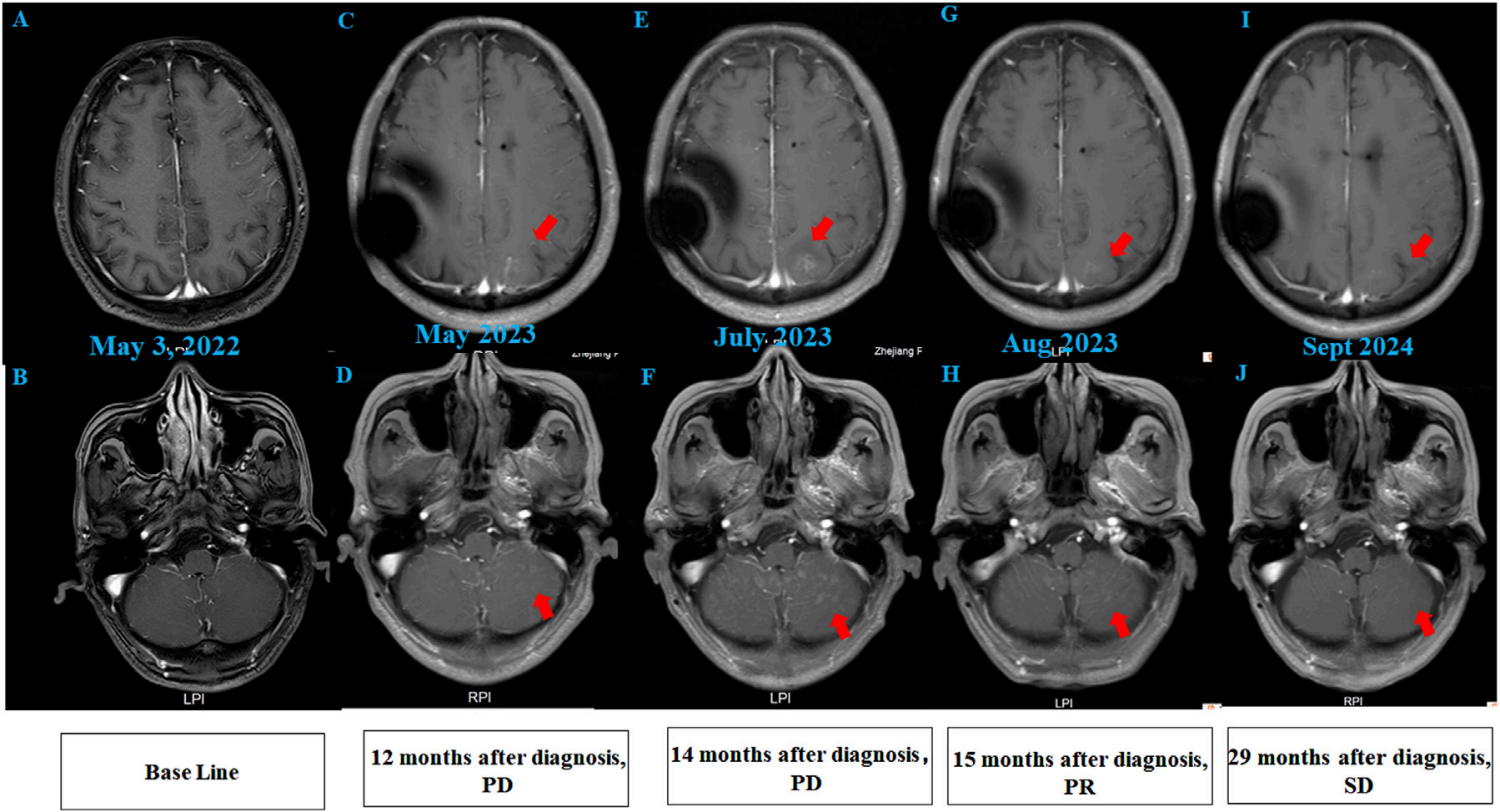
Brain MRI findings of the patient. **(A,B)** Initial brain MRI demonstrated T2/FLAIR hyperintensities in the white matter (Fazekas grade 1) and age-related cerebral atrophy. **(C,D)** MRI at 12 months post-diagnosis (after 12 cycles of intrathecal pemetrexed) revealed new abnormal signal foci in the left frontal lobe, bilateral parietal-occipital lobes, and cerebellum, indicating progressive disease (PD). **(E,F)** Post-whole brain radiotherapy, MRI showed increased abnormal signals compared to prior imaging. **(G,H)** After 2 cycles of intrathecal thiotepa, MRI demonstrated slight regression of metastatic tumors, consistent with partial response (PR). **(I,J)** Follow-up MRI revealed stable disease (SD), with no further progression of lesions.

A 68-year-old chronic smoker presented with a 1-month history of progressive dizziness, memory decline, and appetite loss, initially misdiagnosed as Alzheimer’s disease at a local hospital. Upon admission to our institution in May 2022, neurological examination revealed lethargy and slowed cognition requiring wheelchair assistance, though physical examination showed no focal deficits. Diagnostic workup demonstrated significant abnormalities: serum CEA was markedly elevated (32.1 μg/L, normal ≤5.0 μg/L), while chest CT identified a 1.8 cm spiculated right upper lobe nodule with adjacent ground-glass opacity, highly suspicious for malignancy (Figures 1A,B). Brain MRI initially showed only nonspecific white matter changes (Figures 2A,B). Pathological analysis of CT-guided lung biopsy confirmed moderately differentiated adenocarcinoma with PD-L1 expression (TPS 10%) (Figure 1C) and a KRAS p. G12V mutation at 27.84% variant allele frequency. The lumbar puncture confirmed leptomeningeal involvement with an opening pressure of 220 mmH_2_O and markedly abnormal CSF parameters including elevated albumin (278.4 mg/dL, normal ≤45.0 mg/dL), hypoglycorrhachia (2.43 mmol/L, normal 2.5–4.5 mmol/L), and elevated lactate dehydrogenase (60 U/L, normal 8–32 U/L) and lactate (5.8 mmol/L, normal 0.5–1.7 mmol/L). The cerebrospinal fluid smear examination showed clustered atypical cells, indicating a high possibility of malignancy (Figure 1D). These findings established the definitive diagnosis of stage IVB (pT1cN0M1b) KRAS p. G12V-mutated lung adenocarcinoma with leptomeningeal metastasis.

### 2.2 Treatments

Initial systemic therapy beginning 7 May 2022 with pemetrexed (500 mg/m^2^), bevacizumab (7.5 mg/kg) and carboplatin (AUC 5) paradoxically exacerbated neurological symptoms, prompting urgent multidisciplinary evaluation. This led to placement of a ventriculoperitoneal shunt (Medtronic) and implantation of an Ommaya reservoir (RE-2021, SOPHYSA UBC) on 19 May 2022 to relieve intracranial hypertension and facilitate subsequent intrathecal therapy.

From May to September 2022, induction therapy comprised intrathecal pemetrexed (20 mg on days 1,8) with dexamethasone plus systemic tislelizumab (200 mg on day 1) and carboplatin (AUC 5 on day 1) in every 3 weeks (Q3W). This regimen stabilized pulmonary lesions (Figures 1E,F) and improved neurological status, though grade II thrombocytopenia emerged. Maintenance therapy from October 2022 onward utilized higher-dose intrathecal pemetrexed (40 mg) with intravenous tislelizumab, maintaining response without new toxicity until June 2023.

### 2.3 Disease progression and salvage therapy

After 6 months of maintenance treatment, the patient developed worsening neurological symptoms, including unsteady gait and dizziness. A head MRI revealed multiple abnormal signal foci (Figures 2C,D). Due to confirmed intracranial metastasis, the patient underwent whole brain radiotherapy (WBRT) (3000 cGy/10 fx), but neurological symptoms persisted. CSF cytology detected suspicious cells (Supplementary material online, Supplementary Figure S1), and follow-up MRI showed progression of intracranial lesions, particularly in the left frontal lobe (Figures 2E,F).

Given disease progression, the patient received intrathecal chemotherapy with thiotepa (10 mg) and dexamethasone (5 mg) on days 1, 8, and 15 of a 4-week cycle, alongside intravenous tislelizumab and bevacizumab on day 1. After two cycles, CSF cytology showed no abnormal cells, symptoms improved significantly, and the intracranial lesions slightly regressed (Figures 2G,H). Genetic testing of CSF confirmed a KRAS p.G12V mutation, consistent with the prior lung pathology.

Following 10 cycles of intrathecal thiotepa, the patient developed thrombocytopenia (platelets: 42, normal range: 125–350 × 10^9/L)). Due to concerns over bone marrow suppression, the regimen was switched to intrathecal methotrexate at a reduced frequency—10 mg on days 1 and 4 every three weeks—while continuing intravenous tislelizumab and bevacizumab. The patient tolerated this adjusted regimen well, with stable disease and no new neurological symptoms after two cycles.

**FIGURE 3:**
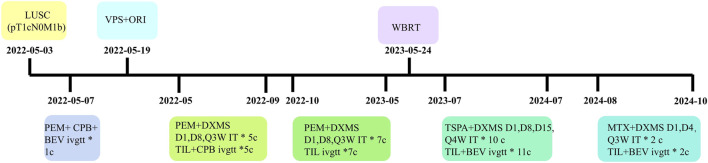
The treatment chart of the patient. LUSC, lung squamous cell carcinoma; VPS, ventriculoperitoneal shunt; ORI, Ommaya reservoir implantation; WBRT, whole brain radiotherapy; PEM, pemetrexed; CPB, carboplatin; TIL, tislelizumab; BEV, bevacizumab; DXMS, dexamethasone; TSPA, Thiotepa; MTX, methotrexate; D, day; Q, every; W, week, c, cycles; ivgtt, intravenous infusion; IT, intrathecal.

To date, the patient has survived for 29 months since LM diagnosis, maintaining a performance status of 1 and a good quality of life with stable pulmonary and intracranial disease on imaging (Figures 1G,H; Figures 2I,J). The treatment summary of the patient is shown in Figure 3.

### 2.4 From the patient’s perspective

The VPS and Ommaya reservoir have significantly facilitated intrathecal chemotherapy, eliminating his fear of lumbar puncture. Furthermore, the attentive care provided by the medical staff has greatly alleviated his anxiety. Consequently, he is overjoyed to see an improvement in his symptoms.

## 3 Discussion and literature review

### 3.1 Clinical manifestations and diagnosis of LM

The clinical manifestations of LM patients are highly diverse, and onset is often insidious. Initial symptoms may include weakness, headaches, back pain, nausea, vomiting, and so on (Ozcan et al., 2023; Wang et al., 2022). In our case, the patient presented with were dizziness, loss of appetite, and memory decline, initially misdiagnosed as Alzheimer’s disease. Due to its nonspecific symptoms, LM is frequently misdiagnosed, delaying optimal treatment. While MRI abnormalities may support LM diagnosis in typical cases (Wang et al., 2022), CSF cytology remains the gold standard. Repeated CSF analyses can achieve up to 90% sensitivity (Thakkar et al., 2020). Our patient exhibited malignant cells in CSF, elevated ICP, and abnormal CSF profiles (elevated protein, low glucose, lymphocytic pleocytosis). These findings—high protein, hypoglycorrhachia, pleocytosis, and positive cytology—are hallmark features of LM (Ozcan et al., 2023).

### 3.2 Current treatment paradigms in LM

Patients with LM face a poor prognosis, with median survival typically ranging from 1 to 4 months. However, certain subgroups, particularly those eligible for molecularly targeted therapies or with radiation-sensitive disease, may achieve better outcomes (Lamba et al., 2021). Current treatment strategies aim to stabilize neurological symptoms and prolong survival (Palmisciano et al., 2022), though standardized protocols remain lacking. The therapeutic arsenal includes whole neuraxial radiation therapy, systemic chemotherapy, and intrathecal administration of traditional chemotherapeutic agents. Notably, molecular targeted therapies have emerged as preferred first-line options for patients with specific actionable mutations. Osimertinib has demonstrated significant survival benefits in NSCLC patients with epidermal growth factor receptor (EGFR) mutations who develop LM (Yang et al., 2020; Lee et al., 2020). Similarly, the third-generation anaplastic lymphoma kinase (ALK) inhibitor Lorlatinib shows excellent central nervous system (CNS) penetration (Bauer et al., 2020). However, for NSCLC patients lacking targetable mutations, treatment options remain limited, highlighting an urgent need for more effective therapeutic approaches.

### 3.3 KRAS G12V mutation in LM

The KRAS mutation occurs in approximately 30% of human cancers (Hung et al., 2020), with most variants located at codon G12. Among these, the G12V subtype accounts for roughly 20% (Harris and Thawani, 2024). In gastric cancer, KRAS G12V is associated with poorer patient survival (Fu et al., 2019). Notably, Fukuda et al. demonstrated in a LCM mouse model that KRAS-G12V-driven, EGFR-mutant lung cancer cells developed Osimertinib resistance within the leptomeningeal space (Fukuda et al., 2021). Currently, no approved targeted therapies exist for KRAS G12V. However, preclinical studies suggest immunotherapy may hold promise (Zhang et al., 2024). In our case, both the patient’s tumor tissue and CSF tested positive for KRAS G12V, leaving no viable targeted treatment options.

### 3.4 Multimodal management of LM

The management of LM from NSCLC includes IC(4), which can be administered either through lumbar puncture or via an Ommaya reservoir. While lumbar puncture remains an option, it often results in poor patient compliance due to procedural discomfort and significant side effects. In contrast, the Ommaya reservoir - a subcutaneous implant with a catheter extending into the lateral ventricle - offers several advantages, including avoidance of repeated lumbar punctures, more consistent drug distribution, and the ability to both administer chemotherapy and sample CSF through a single access point (Zubair and De Jesus, 2024). However, many LM patients develop intracranial hypertension, which can lead to CSF leakage around the Ommaya device and clinical deterioration. To address this, VPS has been employed to effectively reduce ICP. Studies by Kim et al. demonstrate that VPS not only alleviates ICP-related symptoms but may also improve overall survival (Kim et al., 2019). The versatility of VPS allows its use in both communicating and non-communicating hydrocephalus (Kim et al., 2019). Recent evidence from Chen et al. suggests that aggressive shunt placement may be particularly beneficial for LM patients (Chen et al., 2023). The patient’s CSF opening pressure, measured via lateral decubitus lumbar puncture, was 220 mm H_2_O, approximately equivalent to 16.18 mm Hg. A systematic review indicates that the normal range of lumbar CSF opening pressure is between 6.3 and 15.9 mmHg (Norager et al., 2021). Thus, the patient’s pressure clearly exceeded the upper limit of normal. Furthermore, several studies investigating intrathecal chemotherapy and CSF shunting have utilized an inclusion criterion of lumbar puncture opening pressure greater than 20 cm H_2_O (approximately 14.7 mmHg) (Woo et al., 2022; Su et al., 2022). Therefore, based on the available evidence, we considered the measured CSF pressure of 220 mm H_2_O clinically significant and indicative of elevated intracranial pressure, supporting the decision for shunt insertion. A critical consideration in treatment planning is that while VPS effectively controls ICP, it lacks a dedicated channel for chemotherapy administration. Clinical data from Huntoon et al. highlight the importance of combined approaches, showing that patients receiving both VPS and multiple IC sessions achieved significantly longer survival (11.7 months) compared to those without IC (2.8 months) (Huntoon et al., 2024). In the presented case, the combination of VPS with Ommaya reservoir implantation provided dual benefits of ICP control and preserved access for intrathecal therapy, illustrating the potential advantages of this integrated surgical approach.

### 3.5 Selection of IC agents for LM

The most commonly used IC drugs for LM include methotrexate, cytarabine, and thiotepa (Ozcan et al., 2023; Wang et al., 2022), although no single regimen has demonstrated clear superiority (Ozcan et al., 2023). Our review of clinical trials (Fan et al., 2024; Li H. et al., 2023; Fan et al., 2021; Miao et al., 2020; Ju et al., 2016; Pan et al., 2020; Pan et al., 2016) and case reports (Zhong et al., 2024; Li G. et al., 2023; Zheng et al., 2022; Ma et al., 2022; Li et al., 2020; Wang et al., 2018) (shown in Table 1) from the past decade reveals that pemetrexed has become the most frequently employed IC agent for LM secondary to lung cancer, with administered doses ranging from 10 mg to 50 mg. Dose-finding studies have provided important guidance: Pan et al. demonstrated that 10 mg of intrathecal pemetrexed given 1–2 times weekly with vitamin supplementation offered favorable efficacy with manageable toxicity in patients with recurrent LM from lung adenocarcinoma (Pan et al., 2019), while Fan et al.'s phase I study ultimately selected 50 mg as the optimal dose after evaluating a 15–80 mg range (Fan et al., 2024; Fan et al., 2021).

**TABLE 1:** Selected studies on intrathecal chemotherapy in lung cancer patients with leptomeningeal metastasis.

Publication	Type of study	No. of patients	Patient characteristics	Treatment regimen	Means of intrathecal injection	Response to therapy
Fan et al. (2024)	Phase II clinical study (ChiCTR1800016615)	132	NSCLC-LM who progressed from TKI	IP (50 mg × D1, D5 q1w, then q3w×4c, then once monthly)	Lumbar puncture or Ommaya reservoir	mOS: 12 months (95% CI 10.4–13.6 months)
Li et al. H., (2023)	Phase I trial (ChiCTR2000028936)	23	LUAD-LM patients who had progressed after at least two prior treatments	PEM from 30 mg to 50 mg D1, D8 q3w	Ommaya reservoir	mPFS:6.3 months; mOS: 9.5 months
Fan et al. (2021)	Phase 1/2 (ChiCTR1800016615)	30	Patients with EGFR mutant NSCLC-LM who had failed on TKIs	The dose of IP was escalated from 15 mg to 80 mg in a phase 1 study. 50 mg PEM was used in the phase 2 study	Lumbar puncture	mOS: 9.0 months (95%CI 6.6–11.4 months)
Pan et al. (2020)	Phase I/II study (NCT03507244)	34	21 lung cancer, 5 small-cell lung cancer, 4 breast cancer, 4 and others without a history of intra-CSF therapy or WBRT	Induction IP (PEM 10 mg, DXMS 5 mg, q1w ×4c), followed by concomitant IFRT (40 Gy in 20 fractions) within 3 days	Lumbar puncture	mOS: 5.5 (0.3–16.6) mo. Median NPFS: 3.5 (0.3–15.2) mo. 6-month NPFS rate was 47%. 1-year survival rate was 21.6%
Miao et al. (2020)	Single-center retrospective study	23	NSCLC with 16 EGFR mutations, 2 ALK fusions, 1 ROS1 fusion, 1 ERBB2 mutation, and 3 wild-type after first-line standard or TKIs treatment failure	Based on 10 mg IP with multiple therapy (19 TKIs, 10 systemic chemotherapy, 10 antivascular therapy, 1 immunotherapy, 1 WBRT, 13 two or more of the combination treatment modes)	Lumbar puncture or Ommaya reservoir	mPFS:9.6 months [95% CI: 3.4–15.8 months]. OS was not mature at the final follow-up
Pan et al. (2016)	Prospective, single-arm study	59	42 lung cancer, 11 breast cancer and 6 others with at least one poor prognostic factor	IC (MTX 12.5–15 mg and DXM 5 mg, weekly) concomitant IF-RT (whole brain and/or spinal canal RT, 40 Gy/20f)	Lumbar puncture	mOS:6.5 months (0.4–36.7 months),1-year-survival rate was 21.3%
Ju et al. (2016)	UN	20	LUAD	15 patients: IT nimotuzumab (50 mg/week) and MTX (5–10 mg/week); 5 patients: intrathecal nimotuzumab only	UN	mOS:5 months (95% CI 2.4–7.6 months)
Zhong et al. (2024)	Case report	1	Older man, post-thoracoscopic pneumonectomy, diagnosed IIA LUAD with EGFR21 L858R mutation	30 mg IP every 2–3 months, 2–3 times per course (4–6 days each time), and continued 160 mg of Osimertinib	UN	This patient has been alive and well with disease control for 28 months since the diagnosis of meningeal metastases
Li G. et al. (2023)	Case report	1	Case 1:43-year-old man, post-surgery, diagnosed 3A NSCLC with EGFR exon 19 deletion	Case 1:IT MTX and oral Afatinib	UN	Case 1:The patient has shown clinical remission which is longer than 10 months
Zheng et al. (2022)	Case report	1	56-year-old man, post-thoracoscopic resection, diagnosed LUAD with EGFR exon 19 deletion and EGFR-SEPT14 fusions in CSF	Osimertinib combined IP 50 mg D1, D8 q21d	UN	The response was graded as complete remission
Ma et al. (2022)	Case report	1	Female, post-radical surgery, diagnosed IIIA (pT1bN2) LUAD, resistant EGFR exon20ins mutation	IC with 15 mg MTX 6 times + WBRT with SIB + Osimertinib. The first remission lasted 6 months, 15 mg IP 6times + 10 mg/d Anlotinib	Second time IC via Ommaya reservoir	Post-LM OS was 13.5 months
Li et al. (2020)	Case report	1	57-year-old female, diagnosed LUAD with EGFR exon 19 deletion mutations	30 mg IP D1,D8 q3w, combined 160 mg of Osimertinib	Ommaya reservoir	Alleviation of the neurological symptoms, and clearing of CSF cytology
Wang et al. (2018)	Case report	1	38-year-old, diagnosed NSCLC with EGFR L858R mutation	Osimertinib + oral Temozolomide +6 times IC of Cytarabine + WBRT	UN	After 18 months, no recurrence or new lesions have been observed

IC, intrathecal chemotherapy; AEs, adverse events; NSCLC, non-small cell lung cancer; TKIs, tyrosine-kinase inhibitors; PEM, pemetrexed; IP, intrathecal pemetrexed; DXMS, dexamethasone; D/d, day; q, every; c, cycles; w, week; ivgtt, intravenous infusion; IT, intrathecal; mOS, median overall survival; CI, confidence interval; mo, months; LUAD, lung adenocarcinoma; mPFS, median progression-free survival; EGFR, epidermal growth factor receptor; CSF, cerebrospinal fluid; WBRT, whole brain radiotherapy; IFRT, involved-field radiotherapy; NPFS, neurological progression-free survival; ALK, anaplastic lymphoma kinase; ROS1, ROS, proto-oncogene 1; ERBB2, Erb-B2, receptor tyrosine kinase 2; MTX, methotrexate; DXM, dexamethasone; IF-RT, field radiotherapy; UN, unknown; SIB, simultaneous integrated boost.

In our case, intrathecal pemetrexed (40 mg) demonstrated safety and efficacy, achieving >1 year of initial remission when combined with systemic therapy. However, at the time of recurrence, WBRT provided limited benefit. We subsequently transitioned to intrathecal chemotherapy with thiotepa, an alkylating agent known for its excellent CNS penetration and long-standing use since the 1970s. Recent evidence from Jamison et al. (2024) supports thiotepa as a viable alternative, given its favorable efficacy/safety profile. Our patient maintained nearly 1 year of remission following thiotepa treatment. However, moderate thrombocytopenia developed, necessitating a regimen change. The patient has now completed two cycles of intrathecal methotrexate, which was well-tolerated. Given its pharmacokinetic properties (including an 8–10.5 h half-life), methotrexate remains the first-line intrathecal agent for most solid tumors, particularly breast cancer (Jafari et al., 2024; Palmisciano et al., 2022).

### 3.6 International multidisciplinary team consultation

We have invited international scholars to further discuss several issues regarding the diagnosis and treatment of the patient.

Question 1 Is early Ommaya reservoir implantation appropriate for newly diagnosed leptomeningeal metastasis(LM) patients without sensitive gene mutations?

Expert opinion 1: Dr. Rimas V. Lukas,MD, Lou & Jean Malnati Brain Tumor Institute, Robert H. Lurie Comprehensive Cancer Center, Northwestern University, United States.

It is a very reasonable consideration. A number of thought leaders in the field advocate for this, although there is no firm data to support superior outcomes. It can be a practical approach for patients with a good performance status who have a limited likelihood of deriving benefit from systemically administered targeted therapies.

Expert opinion 2: Koji Fukuda, PhD, Cancer Research Institute, Kanazawa University, Japan.

From a research perspective, early placement of an Ommaya reservoir at the time of leptomeningeal metastasis diagnosis is often considered appropriate in EGFR-wildtype NSCLC. Without actionable mutations, treatment options rely on chemotherapy (including intrathecal therapy) rather than targeted drugs. An Ommaya reservoir provides reliable intraventricular access for repeated intrathecal chemotherapy, showing modest efficacy in LM (response rates ∼50% and median survival of 4–6 months in treated patients). While no randomized trials exist, expert consensus suggests that prompt Ommaya placement can facilitate timely therapy and potentially improve symptom control in this setting.

Question 2 To prevent intracranial hypertension, we sometimes combine Ommaya reservoir implantation with ventriculoperitoneal shunt(VPS). What are your thoughts on this approach?

Expert opinion 1: Rimas V. Lukas, MD, Lou & Jean Malnati Brain Tumor Institute, Robert H. Lurie Comprehensive Cancer Center, Northwestern University, United States.

In our clinical practice we do this in some scenarios where there is symptomatic elevation of intracranial pressure. There is clinical data to support superior outcomes with the use of VPS in patients with LM and elevated intracranial pressure. Huntoon, et al. Neuro Oncol Pract. 2023 is one recent example.

Expert opinion 2: Koji Fukuda, PhD, Cancer Research Institute, Kanazawa University, Japan.

Leptomeningeal spread of cancer can impair CSF flow and lead to hydrocephalus with raised intracranial pressure. Combining an Ommaya reservoir with a VP shunt (often with an on-off valve) addresses both issues by enabling intrathecal chemotherapy delivery and providing CSF diversion to prevent intracranial hypertension. Literature reports (including an extensive retrospective study) indicate that patients who receive both CSF diversion and intrathecal chemotherapy survive longer than those managed with a shunt alone. In practice, the shunt valve can be temporarily closed during intrathecal drug administration and opened afterwards, allowing treatment of the LM while controlling pressure-related symptoms. This combined approach has been associated with significant symptom relief and improved quality of life in LM patients with hydrocephalus.

Question 3 Have you used intrathecal chemotherapy for leptomeningeal metastasis patients? If so, what drug and dosage do you commonly use?

Expert opinion 1: Rimas V. Lukas, MD, Lou & Jean Malnati Brain Tumor Institute, Robert H. Lurie Comprehensive Cancer Center, Northwestern University, United States.

Yes. In our clinical practice, for solid tumors we likely most often use topotecan (Groves, et al., 2008). For HER2+ disease we often consider trastuzumab (Wu et al., 2023; Kumthekar et al., 2023).

Expert opinion 2: Koji Fukuda, PhD, Cancer Research Institute, Kanazawa University, Japan.

Common intrathecal chemotherapy agents in solid tumor LM include methotrexate, cytarabine, and thiotepa. Standard intrathecal dosing reported in the literature is typically around 10–15 mg for methotrexate and about 20–50 mg for cytarabine (with ∼50 mg used for the liposomal slow-release form) per administration. Treatment is usually given in an induction phase (for example, intrathecal injections twice weekly for 2–4 weeks) followed by a maintenance phase with dosing every 2 weeks or monthly. Newer intrathecal agents (such as pemetrexed in NSCLC LM) have also been explored in clinical trials, but methotrexate-based regimens remain a frequently reported approach (References: Zhou et al., 2023; Huntoon et al., 2024; Scott et al., 2016).

### 3.7 Limitations of the current case

To our knowledge, this case represents one of the longest reported survival times in an NSCLC patient with LM harboring the KRAS-G12V mutation (or other non-targetable driver mutations). However, several limitations should be noted.

First, while most literature describes IC administration via lumbar puncture or Ommaya reservoir alone, limited data exist on IC combined with a VPS. Moreover critically, we did not perform therapeutic drug monitoring of pemetrexed, thiotepa, or methotrexate in the CSF or plasma for this patient. The CSF diversion effect of VPS complicates drug concentration monitoring, as therapeutic agents may drain into the peritoneal cavity, potentially reducing the exposure of the leptomeningeal space to the chemotherapy. This pharmacokinetic challenge is supported by the literature. Studies have demonstrated that standard VPS can siphon intraventricularly administered drugs away from the CSF space, leading to underdosing (Huntoon et al., 2024). However, the combination of VPS with an Ommaya reservoir, particularly one equipped with an on-off valve as used in our case, is specifically designed to mitigate this issue by allowing temporary occlusion of the shunt during drug administration (Huntoon et al., 2024). Here, we determined dosing based on published efficacy and safety profiles from studies in the literature, but the precise pharmacokinetic impact of the VPS with on-off valve configuration on the IC agents used warrants further study.

Second, the patient received multiple simultaneous interventions (intrathecal agents, systemic immunotherapy, bevacizumab, WBRT, and VPS), making it impossible to attribute survival benefit to any single component. Our report is hypothesis-generating and underscores the need for prospective studies to delineate the individual contribution of each modality.

Third, the initial diagnostic work-up was incomplete: only PD-L1 immunohistochemistry was performed on the lung biopsy, whereas a full immunohistochemical panel and molecular profiling were omitted for cost-saving reasons. This deficiency may have limited the precision of subtype classification and subsequent therapy selection, highlighting the need for more comprehensive pathological evaluation in future cases.

## 4 Conclusion

We report a NSCLC patient with KRAS p. G12V mutation and LM who achieved 29 months of remission using intrathecal pemetrexed, thiotepa, and methotrexate via an Ommaya reservoir, combined with tislelizumab and bevacizumab. This combined approach shows promise for LM treatment. However, prospective studies are needed to confirm its efficacy and safety.

## Data Availability

The original contributions presented in the study are included in the article/Supplementary Material, further inquiries can be directed to the corresponding author.

## Abbreviations

AE, adverse event; ALK, anaplastic lymphoma kinase; AUC, area under the curve; CNS, central nervous system; CSF, cerebrospinal fluid; EGFR, epidermal growth factor receptor; IC, intrathecal chemotherapy; ICP, intracranial pressure; IP, intrathecal pemetrexed; KRAS, Kirsten rat sarcoma; LCM, leptomeningeal carcinomatosis; LDH, lactate dehydrogenase; LM, leptomeningeal metastasis; LUAD, lung adenocarcinoma; MDT, multi-disciplinary treatment; MRI, magnetic resonance imaging; NSCLC, non-small cell lung cancer; ORI, Ommaya reservoir implantation; OS, overall survival; TKIs, tyrosinekinase inhibitors; VPS, ventriculoperitoneal shunt; WBRT, whole brain radiotherapy.

## References

[B1] Bauer T. M. Shaw A. T. Johnson M. L. Navarro A. Gainor J. F. Thurm H. (2020). Brain penetration of lorlatinib: cumulative incidences of Cns and non-Cns progression with lorlatinib in patients with previously treated Alk-Positive non-small-cell lung cancer. Target Oncol. 15 (1), 55–65. 10.1007/s11523-020-00702-4 32060867 PMC7028836

[B2] Chen K. Y. Wu S. G. Lai D. M. Kuo L. T. Huang A. P. (2023). Multidisciplinary management of patients with non-small cell lung cancer with leptomeningeal metastasis in the tyrosine kinase inhibitor era. J. Neurosurg. 138 (6), 1552–1560. 10.3171/2022.8.Jns221175 36208438

[B3] Fan C. Zhao Q. Li L. Shen W. Du Y. Teng C. (2021). Efficacy and safety of intrathecal pemetrexed combined with dexamethasone for treating tyrosine kinase inhibitor-failed leptomeningeal metastases from Egfr-mutant Nsclc—a prospective, open-label, single-arm phase 1/2 clinical trial (unique identifier: Chictr1800016615). J. Thorac. Oncol. 16 (8), 1359–1368. 10.1016/j.jtho.2021.04.018 33989780

[B4] Fan C. Jiang Z. Teng C. Song X. Li L. Shen W. (2024). Efficacy and safety of intrathecal pemetrexed for Tki-Failed leptomeningeal metastases from Egfr+ nsclc: an expanded, single-arm, phase Ii clinical trial. ESMO Open 9 (4), 102384. 10.1016/j.esmoop.2024.102384 38377785 PMC11076967

[B5] Fu X. H. Chen Z. T. Wang W. H. Fan X. J. Huang Y. Wu X. B. (2019). Kras G12v mutation is an adverse prognostic factor of Chinese gastric cancer patients. J. Cancer 10 (4), 821–828. 10.7150/jca.27899 30854087 PMC6400811

[B6] Fukuda K. Otani S. Takeuchi S. Arai S. Nanjo S. Tanimoto A. (2021). Trametinib overcomes Kras-G12v-Induced osimertinib resistance in a leptomeningeal carcinomatosis model of Egfr-Mutant lung cancer. Cancer Sci. 112 (9), 3784–3795. 10.1111/cas.15035 34145930 PMC8409422

[B41] Groves M.D. Glantz M. J. Chamberlain M.C. Baumgartner K. E. Conrad C. A. Hsu S. (2008). A multicenter phase ii trial of intrathecal topotecan in patients with meningeal malignancies. Neuro Oncol. 10 (02), 208–15. 10.1215/15228517-2007-059 18316473 PMC2613823

[B7] Harris E. Thawani R. (2024). Current perspectives of kras in non-small cell lung cancer. Curr. Probl. Cancer 51, 101106. 10.1016/j.currproblcancer.2024.101106 38879917

[B8] Hung P. S. Huang M. H. Kuo Y. Y. Yang J. C. (2020). The inhibition of wnt restrain Kras(G12v)-Driven metastasis in non-small-cell lung cancer. Cancers (Basel) 12 (4), 837. 10.3390/cancers12040837 32244355 PMC7226522

[B9] Huntoon K. M. Gasco J. Glitza Oliva I. C. Ferguson S. D. Majd N. K. McCutcheon I. E. (2024). Ventriculoperitoneal shunting with an on-Off valve for patients with leptomeningeal metastases and intracranial hypertension. Neurooncol Pract. 11 (1), 56–63. 10.1093/nop/npad056 38222058 PMC10785578

[B10] Jafari F. Nodeh M. M. Hosseinjani H. Baharara H. Azad S. Arasteh O. (2024). A review on the efficacy and safety of intrathecal administration of novel medications for leptomeningeal metastases in solid cancers. Curr. Med. Chem. 31 (19), 2732–2750. 10.2174/0929867330666230508142657 37157199

[B11] Jamison T. Haque E. Muhsen I. N. Samarkandi H. Fakih R. E. Aljurf M. (2024). Revisiting intrathecal thiotepa: efficacy and safety in secondary cns malignancies. Med. Oncol. 41 (7), 177. 10.1007/s12032-024-02401-w 38884819

[B12] Ju Y. Wang J. Sun S. Jiao S. (2016). Nimotuzumab treatment and outcome analysis in patients with leptomeningeal metastasis from nonsmall cell lung cancer. J. Cancer Res. Ther. 12 (Suppl. ment), C181–C185. 10.4103/0973-1482.200596 28230014

[B13] Kim H. S. Park J. B. Gwak H. S. Kwon J. W. Shin S. H. Yoo H. (2019). Clinical outcome of cerebrospinal fluid shunts in patients with leptomeningeal carcinomatosis. World J. Surg. Oncol. 17 (1), 59. 10.1186/s12957-019-1595-7 30917830 PMC6438037

[B43] Kumthekar P. C. Avram M. J. Lassman A. B. Lin N. U. Lee E. Grimm S. A. (2023). A phase I/Ii study of intrathecal trastuzumab in human epidermal growth factor receptor 2-positive (Her2-positive) cancer with leptomeningeal metastases: safety, efficacy, and cerebrospinal fluid pharmacokinetics. Neuro Oncol. 25 (03), 557–65. 10.1093/neuonc/noac195 35948282 PMC10013631

[B14] Lamba N. Wen P. Y. Aizer A. A. (2021). Epidemiology of brain metastases and leptomeningeal disease. Neuro Oncol. 23 (9), 1447–1456. 10.1093/neuonc/noab101 33908612 PMC8408881

[B15] Lee J. Choi Y. Han J. Park S. Jung H. A. Su J. M. (2020). Osimertinib improves overall survival in patients with Egfr-Mutated nsclc with leptomeningeal metastases regardless of T790m mutational status. J. Thorac. Oncol. 15 (11), 1758–1766. 10.1016/j.jtho.2020.06.018 32652216

[B16] Li H. Lin Y. Yu T. Xie Y. Feng J. Huang M. (2020). Treatment response to intrathecal chemotherapy with pemetrexed Via an ommaya reservoir in Egfr-Mutated leptomeningeal metastases from non-small cell lung cancer: a case report. Ann. Palliat. Med. 9 (4), 2341–2346. 10.21037/apm-19-521 32648459

[B17] Li H. Zheng S. Lin Y. Yu T. Xie Y. Jiang C. (2023). Safety, pharmacokinetic and clinical activity of intrathecal chemotherapy with pemetrexed Via the ommaya reservoir for leptomeningeal metastases from lung adenocarcinoma: a prospective phase I study. Clin. Lung Cancer 24 (2), e94–e104. 10.1016/j.cllc.2022.11.011 36588048

[B18] Li G. G. Fang M. Zhou Y. Liu X. Tian P. Mei F. (2023). Afatinib overcoming resistance to icotinib and osimertinib in nsclc with leptomeningeal metastasis in patients with acquired Egfr L858r/T790m or L858r/S768i mutations: two case reports. Heliyon 9 (10), e20690. 10.1016/j.heliyon.2023.e20690 37860534 PMC10582297

[B19] Ma Y. Liu H. Zhang M. Liu B. Ding Q. Zhang L. (2022). Successful treatment using targeted therapy, radiotherapy, and intrathecal chemotherapy in a patient with leptomeningeal metastasis with an epidermal growth factor receptor Exon 20 insertion mutation: a case report. Ann. Palliat. Med. 11 (4), 1533–1541. 10.21037/apm-21-321 34263612

[B20] Miao Q. Zheng X. Zhang L. Jiang K. Wu B. Lin G. (2020). Multiple combination therapy based on intrathecal pemetrexed in non-small cell lung cancer patients with refractory leptomeningeal metastasis. Ann. Palliat. Med. 9 (6), 4233–4245. 10.21037/apm-20-2086 33302683

[B21] Norager N. H. Olsen M. H. Pedersen S. H. Riedel C. S. Czosnyka M. Juhler M. (2021). Reference values for intracranial pressure and lumbar cerebrospinal fluid pressure: a systematic review. Fluids Barriers CNS 18 (1), 19. 10.1186/s12987-021-00253-4 33849603 PMC8045192

[B22] Ozcan G. Singh M. Vredenburgh J. J. (2023). Leptomeningeal metastasis from non-small cell lung cancer and current landscape of treatments. Clin. Cancer Res. 29 (1), 11–29. 10.1158/1078-0432.Ccr-22-1585 35972437

[B23] Palmisciano P. Watanabe G. Conching A. Ogasawara C. Vojnic M. D'Amico R. S. (2022). Intrathecal therapy for the management of Leptomeningeal metastatic disease: a scoping review of the current literature and ongoing clinical trials. J. Neurooncol 160 (1), 79–100. 10.1007/s11060-022-04118-0 35999434

[B24] Pan Z. Yang G. He H. Zhao G. Yuan T. Li Y. (2016). Concurrent radiotherapy and intrathecal methotrexate for treating leptomeningeal metastasis from solid tumors with adverse prognostic factors: a prospective and single‐arm study. Int. J. Cancer 139 (8), 1864–1872. 10.1002/ijc.30214 27243238 PMC5096248

[B25] Pan Z. Yang G. Cui J. Li W. Li Y. Gao P. (2019). A pilot phase 1 study of intrathecal pemetrexed for refractory leptomeningeal metastases from non-small-cell lung cancer. Front. Oncol. 9, 838. 10.3389/fonc.2019.00838 31544065 PMC6730526

[B26] Pan Z. Yang G. He H. Cui J. Li W. Yuan T. (2020). Intrathecal pemetrexed combined with involved-field radiotherapy as a first-line intra-csf therapy for leptomeningeal metastases from solid tumors: a phase I/Ii study. Ther. Adv. Med. Oncol. 12, 1758835920937953. 10.1177/1758835920937953 32733606 PMC7370561

[B40] Scott B. J. Oberheim-Bush N. A. Kesari S. (2016). Leptomeningeal metastasis in breast cancer - a systematic review. Oncotarget 7 (04), 3740–3747. 10.18632/oncotarget.5911 26543235 PMC4826166

[B27] Su Y. H. Chiang C. L. Yang H. C. Hu Y. S. Chen Y. W. Luo Y. H. (2022). Cerebrospinal fluid diversion and outcomes for lung cancer patients with leptomeningeal carcinomatosis. Acta Neurochir. (Wien) 164 (2), 459–467. 10.1007/s00701-021-04763-w 33646444

[B28] Thakkar J. P. Kumthekar P. Dixit K. S. Stupp R. Lukas R. V. (2020). Leptomeningeal metastasis from solid tumors. J. Neurol. Sci. 411, 116706. 10.1016/j.jns.2020.116706 32007755

[B29] Wang Y. Liu S. Wei X. Yan B. Li J. Su Z. (2018). Non-small cell lung cancer leptomeningeal metastases treated with intrathecal therapy plus osimertinib and temozolomide and whole-brain radiation therapy: a case report. Onco Targets Ther. 11, 4733–4738. 10.2147/ott.S164968 30127621 PMC6091472

[B30] Wang Y. Yang X. Li N. J. Xue J. X. (2022). Leptomeningeal metastases in non-small cell lung cancer: diagnosis and treatment. Lung Cancer 174, 1–13. 10.1016/j.lungcan.2022.09.013 36206679

[B31] Woo B. Gwak H. S. Kwon J. W. Shin S. H. Yoo H. (2022). Lumboperitoneal shunt combined with ommaya reservoir enables continued intraventricular chemotherapy for leptomeningeal metastasis with increased intracranial pressure. Brain Tumor Res. Treat. 10 (4), 237–243. 10.14791/btrt.2022.0022 36347638 PMC9650125

[B42] Wu S. A. Jia D. T. Schwartz M. Mulcahy M. Guo K. Tate M. C. (2023). Her2+ esophageal carcinoma leptomeningeal metastases treated with intrathecal trastuzumab regimen. CNS Oncol. 12 (03), Cns99. 10.2217/cns-2022-0018 37219390 PMC10410688

[B32] Wu Y. L. Zhou L. Lu Y. (2016). Intrathecal chemotherapy as a treatment for leptomeningeal metastasis of non-small cell lung cancer: a pooled analysis. Oncol. Lett. 12 (2), 1301–1314. 10.3892/ol.2016.4783 27446430 PMC4950629

[B33] Yang J. C. H. Kim S. W. Kim D. W. Lee J. S. Cho B. C. Ahn J. S. (2020). Osimertinib in patients with epidermal growth factor receptor mutation-positive non-small-cell lung cancer and leptomeningeal metastases: the bloom study. J. Clin. Oncol. 38 (6), 538–547. 10.1200/jco.19.00457 31809241 PMC7030895

[B34] Zhang M. Xu W. Luo L. Guan F. Wang X. Zhu P. (2024). Identification and affinity enhancement of T-Cell receptor targeting a Kras(G12v) cancer neoantigen. Commun. Biol. 7 (1), 512. 10.1038/s42003-024-06209-2 38684865 PMC11058820

[B35] Zheng S. Li H. Feng J. Jiang C. Lin Y. Xie Y. (2022). Complete remission in leptomeningeal metastasis of nsclc with rare Egfr-Sept14 fusion treated with osimertinib combined with intrathecal chemotherapy with pemetrexed. Anticancer Drugs 33 (1), e795–e798. 10.1097/cad.0000000000001222 34486539

[B36] Zhong W. Wu L. Huang L. Wang J. Shi H. Wu S. (2024). Double-dose osimertinib combined with intrathecal injection of pemetrexed improves the efficacy of Egfr-Mutant non-small cell lung cancer and leptomeningeal metastasis: case report and literature review. Front. Oncol. 14, 1377451. 10.3389/fonc.2024.1377451 38711856 PMC11070505

[B38] Zhou T. Zhu S. Xiong Q. Gan J. Wei J. Cai J. (2023). Intrathecal chemotherapy combined with systemic therapy in patients with refractory leptomeningeal metastasis of non-small cell lung cancer: a retrospective study. BMC Cancer 23 (01), 33. 10.1186/s12885-023-10806-5 37041504 PMC10088274

[B37] Zubair A. De Jesus O. (2024). Ommaya reservoir. Treasure Island (FL): StatPearls Publishing LLC. 32644437

