# Supplementary material for: Case Report: Extended survival in KRAS-G12V NSCLC with leptomeningeal metastasis through integrated intrathecal chemotherapy and systemic therapies
Source: Front Pharmacol. 2025 Oct 22;16:1632369. doi: 10.3389/fphar.2025.1632369 (PMC12585955; doi:10.3389/fphar.2025.1632369)
Supplement: Supplementary file 1 [file DataSheet1.pdf]

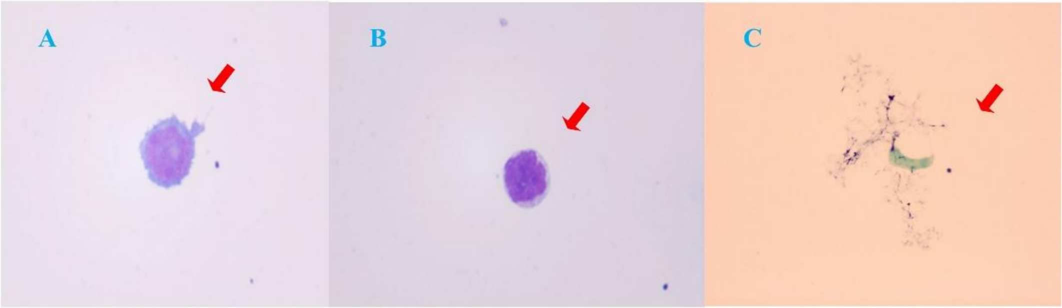

Figure S1. Serial cerebrospinal fluid (CSF) findings during disease progression.

(A) Routine CSF cytology: scant nucleated cells (predominantly macrophages with occasional lymphocytes); a single suspicious cell is present (arrow) (25 July 2023, 100× oil).

(B) Routine CSF cytology: macrophages visible; one atypical cell (arrow) suggestive of tumor cell (1 August 2023, 100× oil).

(C) CSF pathological liquid-based smear: rare degenerated, atypical epithelioid cells confirming malignant involvement (28 August 2023, 100×).
